# Evaluation of the MeroRisk Calculator, A User-Friendly Tool to Predict the Risk of Meropenem Target Non-Attainment in Critically Ill Patients

**DOI:** 10.3390/antibiotics10040468

**Published:** 2021-04-20

**Authors:** Uwe Liebchen, Marian Klose, Michael Paal, Michael Vogeser, Michael Zoller, Ines Schroeder, Lisa Schmitt, Wilhelm Huisinga, Robin Michelet, Johannes Zander, Christina Scharf, Ferdinand A. Weinelt, Charlotte Kloft

**Affiliations:** 1 Department of Clinical Pharmacy and Biochemistry, Institute of Pharmacy, Freie Universität Berlin, Kelchstr. 31, 12169 Berlin, Germany; Uwe.Liebchen@med.uni-muenchen.de (U.L.); marian.klose@fu-berlin.de (M.K.); lisa.ehmann@fu-berlin.de (L.S.); robin.michelet@fu-berlin.de (R.M.); ferdinand.weinelt@fu-berlin.de (F.A.W.); 2 Department of Anaesthesiology, University Hospital, LMU Munich, Marchioninistr. 15, 81377 Munich, Germany; michael.zoller@med.uni-muenchen.de (M.Z.); Ines.Schroeder@med.uni-muenchen.de (I.S.); Christina.Scharf@med.uni-muenchen.de (C.S.); 3 Institute of Laboratory Medicine, University Hospital, LMU Munich, Marchioninistr. 15, 81377 Munich, Germany; Michael.Paal@med.uni-muenchen.de (M.P.); Michael.Vogeser@med.uni-muenchen.de (M.V.); j.zander@labor-brunner.de (J.Z.); 4 Graduate Research Training Program PharMetrX, Freie Universität Berlin, 12169 Berlin, Germany; 5 Graduate Research Training Program PharMetrX, Universität Potsdam, 14476 Potsdam, Germany; 6 Institute of Mathematics, Universität Potsdam, Karl-Liebknecht-Str. 24-25, 14476 Potsdam, Germany; huisinga@uni-potsdam.de; 7 Laboratory Dr. Brunner, Luisenstr. 7e, 78464 Konstanz, Germany

**Keywords:** risk assessment, excel tool, individualized dosing, model-based evaluation

## Abstract

Background: The MeroRisk-calculator, an easy-to-use tool to determine the risk of meropenem target non-attainment after standard dosing (1000 mg; q8h), uses a patient’s creatinine clearance and the minimum inhibitory concentration (MIC) of the pathogen. In clinical practice, however, the MIC is rarely available. The objectives were to evaluate the MeroRisk-calculator and to extend risk assessment by including general pathogen sensitivity data. Methods: Using a clinical routine dataset (155 patients, 891 samples), a direct data-based evaluation was not feasible. Thus, in step 1, the performance of a pharmacokinetic model was determined for predicting the measured concentrations. In step 2, the PK model was used for a model-based evaluation of the MeroRisk-calculator: risk of target non-attainment was calculated using the PK model and agreement with the MeroRisk-calculator was determined by a visual and statistical (Lin’s concordance correlation coefficient (CCC)) analysis for MIC values 0.125–16 mg/L. The MeroRisk-calculator was extended to include risk assessment based on EUCAST-MIC distributions and cumulative-fraction-of-response analysis. Results: Step 1 showed a negligible bias of the PK model to underpredict concentrations (−0.84 mg/L). Step 2 revealed a high level of agreement between risk of target non-attainment predictions for creatinine clearances >50 mL/min (CCC = 0.990), but considerable deviations for patients <50 mL/min. For 27% of EUCAST-listed pathogens the median cumulative-fraction-of-response for the observed patients receiving standard dosing was < 90%. Conclusions: The MeroRisk-calculator was successfully evaluated: For patients with maintained renal function it allows a reliable and user-friendly risk assessment. The integration of pathogen-based risk assessment substantially increases the applicability of the tool.

## 1. Introduction

Prevalence and mortality rates are high in critically ill patients with severe infections [1,2,3]. Effective antibiotic therapy (appropriate activity spectrum and adequate drug exposure) plays a key role in the treatment of severe infections, e.g., in patients with sepsis every hour of delayed therapy initiation increases the mortality [4,5,6]. Particularly in critically ill patients, it is challenging to select an appropriate dosing regimen resulting in adequate antibiotic drug exposure. The pathophysiological changes observed in critically ill patients often lead to pharmacokinetic alterations and—if doses are not adjusted—to suboptimal drug exposure [7,8,9]. How to predict those changes and adapt dosing to achieve optimal antibiotic efficacy has been at the center of ongoing discussions [10,11]. Numerous reports of subtherapeutic concentrations of beta-lactams, especially in patients with hyperdynamic kidney function, highlight the importance of detecting patients at risk of subtherapeutic concentrations and call for a targeted optimization of dosing [12,13,14,15,16].

Meropenem, a beta-lactam antibiotic covering a broad spectrum of pathogens and exerting a bactericidal mechanism of action, is often used for empirical antibiotic therapy in critically ill patients [17]. The time during which the unbound meropenem concentration remains above the minimal inhibitory concentration (fT > MIC) has been related to therapeutic success [7,18]. However, 54% of the intensive care clinicians responding to a recent survey reported not obtaining any MIC results from their laboratories [19]. Consequently, if the MIC value is not available, the risk of therapeutic failure needs to be assessed based on alternative targets like pathogen-unspecific breakpoints (e.g., EUCAST S/I-breakpoint (susceptible/susceptible, increased exposure)) or general pathogen sensitivity information (e.g., MIC value distribution).

As an attractive option to assess the risk of therapeutic failure, therapeutic drug monitoring (TDM) can be used to effectively optimize target attainment [20,21]. Unfortunately, in many healthcare institutions, TDM of meropenem is rarely available to the attending physician [19,22]. Long TDM turnaround times further limit the possibility of prompt dose adjustments. Therefore, a reliable approach to assess initial dosing based on patient characteristics and knowledge about the pathogen would be a valuable advantage. Mathematical models characterizing the pharmacokinetics of a drug integrated into user-friendly tools or software can help to assess and adjust dosing at the point of care. In 2017, Ehmann et al. developed the MeroRisk-Calculator, an easy-to-use Excel-based tool for estimating the risk of target non-attainment of an individual patient receiving meropenem standard dosing (target: 100% T > MIC) [11]. The MeroRisk-Calculator is based on a regression analysis between the creatinine clearance according to Cockcroft-Gault (CLCRCG) [23] and the minimum meropenem concentrations collected in a controlled clinical trial. Using this approach, the risk assessment is immediate and requires no special technical expertise. Abdul-Aziz et al. referred to the MeroRisk-Calculator as a “promising tool” [10] but suggested a validation of the MeroRisk-Calculator in a routine clinical setting to increase trust in the provided risk predictions. So far, the majority of available tools have not been evaluated in a real-world scenario, lowering trust in their predictions and hindering their implementation [24].

Therefore, this study aims to (i) evaluate the performance of the MeroRisk-Calculator using routine clinical data independent from the development of the tool and (ii) to extend the risk predictions of the MeroRisk-Calculator to include pathogen sensitivity information in case no individual MIC value is available.

## 2. Results

### 2.1. Data and Patients

Included in the analysis were 891 meropenem TDM samples from 155 patients. In Table 1, a summary of patient characteristics for the evaluation dataset is displayed. The patients were predominantly male (65.2%) and had a median creatinine clearance of 86.4 mL/min (5th–95th percentile: 35.4–161 mL/min). For each observed dosing interval, a single meropenem sample was taken, for most patients multiple dosing intervals were observed (mean samples per patient 5.7) and samples were taken at variable time points (median time after last dose: 6.2 h, 5th–95th percentile: 3.72–8.13 h).

### 2.2. Evaluation Step 1: Evaluation of the Potential of the Selected Meropenem Population Pharmacokinetic (PK) Model to Predict the Clinical Routine Dataset

The comparison of the observed meropenem concentrations (evaluation dataset) with the concentrations predicted by the PK model revealed a bias of −0.84 mg/L (−16%), i.e., the PK model slightly underpredicted the measured concentrations. The 50% prediction error interval ranged from −5.0 mg/L (−59%) to 1.2 mg/L (32%) (Supplementary Materials Figure A1). The PK model adequately predicted the observed clinical routine data, and the model was subsequently used to evaluate the MeroRisk-Calculator in step 2 of the evaluation process.

### 2.3. Evaluation Step 2: Evaluation of the MeroRisk-Calculator Based on PK Model Predictions of Meropenem Concentrations 8 h after Dosing

**Figure 1:**
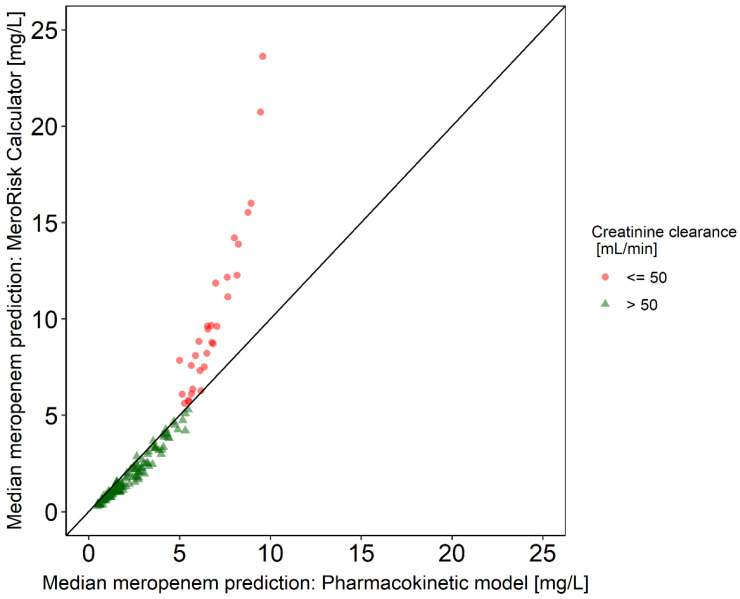
Median meropenem concentrations 8 h after dose predicted by pharmacokinetic model and MeroRisk-Calculator. Median predictions (PK model: stochastic simulations (*n* = 2000), MeroRisk-Calculator: classic theory of linear models [11]) for patients (*n* = 124) with creatinine clearance calculated using Cockcroft–Gault Equation (CLCRCG) > 50 mL/min (green triangles) and patients (*n* = 31) with CLCRCG ≤ 50 mL/min (red points) 8 h after standard dose (1 g meropenem, 0.5 h infusion). Line: Line of identity.

The comparison of median predicted concentrations 8 h after dose (Figure 1) showed a very good agreement for patients with a CLCRCG above 50 mL/min (green triangles). Below 50 mL/min (red points) the MeroRisk-Calculator predicted higher concentrations compared to the PK model.

**Figure 2:**
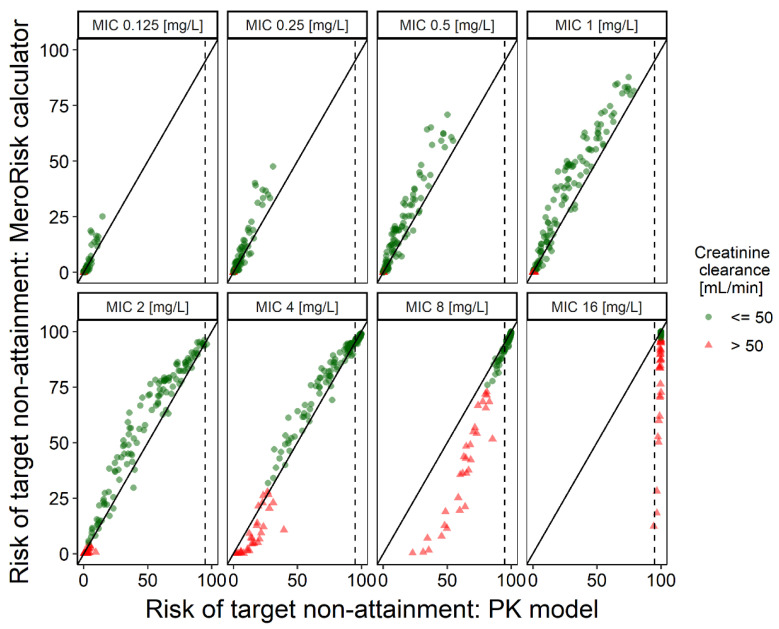
Risk of target non-attainment predicted by MeroRisk-Calculator and by pharmacokinetic (PK) model. The risk of target non-attainment (unbound drug concentration below the minimum inhibitory concentration (MIC) 8 h after standard dose (1 g meropenem, 0.5 h infusion)) was assessed for 155 critically ill patients and selected minimum inhibitory concentrations. Solid line: Line of identity, dashed line: 95% risk predicted by the PK model.

The risk of target non-attainment was predicted for 8 MIC levels ranging from 0.125 mg/L to 16 mg/L (Figure 2) using both the MeroRisk-Calculator and the PK model. The graphical comparison revealed overall good agreement between the two methods; only for MIC values of 8 mg/L and 16 mg/L were risk predictions by the MeroRisk-Calculator biased towards lower risks compared to the evaluated PK model. It has to be noted that for these two high MIC values, the risk of target non-attainment for most patients was predicted to be very high (patients with PK model predicted risk above 95%:65.8% (MIC = 8 mg/L), 100% (MIC = 16 mg/L)). Thus, only a very small fraction of patients with a creatinine clearance below 50 mL/min (*n* = 31, red triangles in Figure 2) led to this strong deviation in the graphical analysis.

The numerical analysis using Lin’s concordance correlation coefficient (CCC) confirmed the visual graphical analysis. The lower one-sided 95% confidence limit of the calculated CCC value for all investigated MIC values was 0.98 and therefore, according to the interpretation by McBride, agreement between the risk predictions of the PK model and the MeroRisk-Calculator was substantial [25]. Including only patients with a CLCRCG above 50 mL/min in the analysis considerably improved the overall agreement shown by an increased CCC value for all MIC values. A full overview of CCC values for different MIC values can be found in Table 2.

### 2.4. Extending Risk Predictions to Include General Pathogen Sensitivity Data

**Figure 3:**
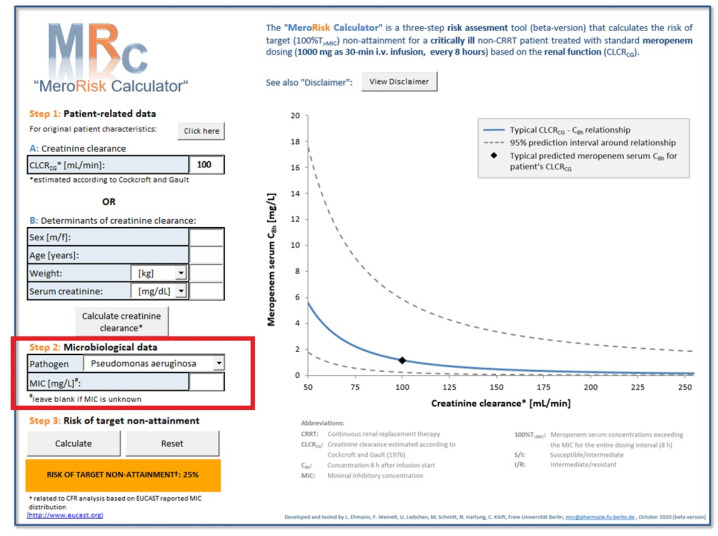
Graphical user interface of the extended MeroRisk-Calculator after risk calculation. Example for illustration: Patient-related and microbiological data: patients with creatinine clearance of 100 mL/min infected with Pseudomonas aeruginosa and no MIC value available. Red box: extended input possibilities for the microbiological data compared to the first version of the MeroRisk-Calculator. Abbreviations: CLCR_CG_, Creatinine clearance estimated according to Cockcroft and Gault equation [23]; CRRT, Continuous renal replacement therapy; C8h, Meropenem serum concentration 8 h after infusion start; MIC, Minimum inhibitory concentration.

The extended version of the MeroRisk-Calculator containing a feature to assess risk based on the selected pathogen is provided as Supplementary Materials B. The tool is compatible with Windows operating systems and Excel version 2010 and newer. Due to changing MIC distributions an up-to-date version of the MeroRisk-Calculator integrating the latest MIC distributions reported by EUCAST can be found online (https://www.bcp.fu-berlin.de/en/pharmazie/faecher/klinische_pharmazie/arbeitsgruppe_kloft/forschung/MRc, (accessed on 16 April 2021)). In each version of the MeroRisk-Calculator, the origin of the employed MIC distribution data is displayed to the user during risk assessment. The intuitive user interface of the MeroRisk-Calculator remained unchanged by the update (Figure 3). Either the CLCRCG or its determinants (sex, age, total body weight, serum creatinine concentration) of a patient need to be provided for risk assessment. In the extended version, all information about the pathogen becoming available over the course of antibiotic treatment are used. If both the pathogen and its MIC value are unknown at therapy start, the MIC entry remains blank and the pathogen entry, “unknown”. In this case risk calculations are based on EUCAST MIC breakpoints chosen by the user. If the pathogen is known but its MIC value is unknown, the respective pathogen can be selected from the dropdown menu. In this case the risk of target non-attainment for the patient is calculated based on CFR analysis and the EUCAST MIC distribution of the selected pathogen [26]. If both pathogen and MIC values are known, they are entered into the MeroRisk-Calculator and risk calculations are based on the provided MIC value. The result of the risk assessment for target non-attainment are displayed in the originally color-coded box (green ≤ 10%, orange > 10% to ≤ 50%, red > 50%) and a graphical illustration of the relationship between CLCRCG and minimum meropenem concentration 8 h after standard dose including the 95% prediction interval is provided.

**Figure 4:**
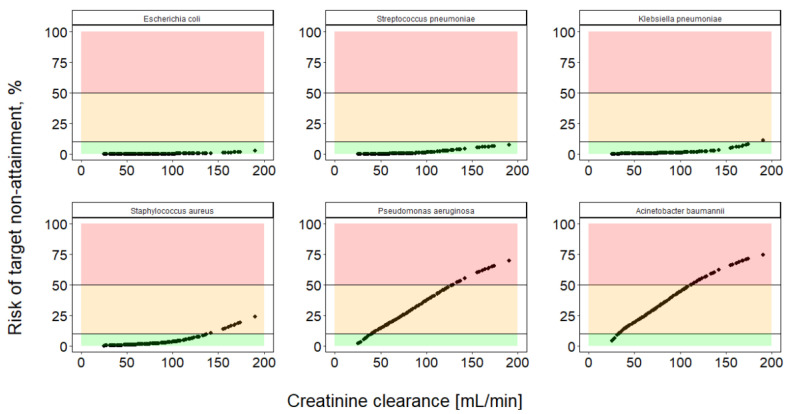
MeroRisk-Calculator predicted risk of target non-attainment for 6 clinically relevant pathogens. The risk of target non-attainment (unbound drug concentration 8 h after standard meropenem dosing below the minimum inhibitory concentration (MIC)) was assessed for critically ill patents (*n* = 155) using EUCAST MIC distributions of the investigated pathogens and cumulative fraction of response analysis. Risk predictions ≤10% (green), >10% to ≤50% (orange) and >50% (red).

In Figure 4 the risk predictions of the MeroRisk-Calculator for six selected clinically relevant pathogens and 155 critically ill patients are presented. Higher creatinine clearances were linked to higher risks of target non-attainment. For susceptible pathogens like Escherichia coli or Streptococcus pneumoniae, the risk of target non-attainment after standard dose was found to be low. More resistant pathogens like Pseudomonas aeruginosa or Acinetobacter baumannii displayed higher risks of target non-attainment for the majority of the investigated patients. Detailed results for all pathogens in the EUCAST database are listed in the Supplementary Materials Figures C1–C5. Overall risk of target non-attainment predicted by the MeroRisk-Calculator was found to be low for most pathogens and the investigated 155 critically ill patients: 73.0% of pathogens revealed median risks below 10%, 18.9% between 10% and 50% and only 8.1% above 50%.

## 3. Discussion

In the presented work we successfully evaluated the MeroRisk-Calculator as a dosing assessment tool in critically ill patients and extended its functionality to integrate risk assessment for target non-attainment based on general pathogen sensitivity data. While there are promising examples of model-informed tools improving drug therapy [27,28], implementation into real-world clinical settings is still lagging behind [29]. The growing repertoire of published PK models is seldom integrated into user-friendly tools and therefore remains inaccessible for most healthcare professionals [30]. In addition, a majority of the available tools have not been evaluated in a real-world scenario, which lowers trust in the reliability of their predictions and further hinders implementation [24]. This investigation is an example of how to evaluate and expand an already published model-informed tool using routine clinical data.

The direct evaluation of the MeroRisk-Calculator was not feasible with our real-world clinical dataset due to the associated variability in sampling times. Instead, a two-step data- and model-based evaluation was chosen allowing the inclusion of a large number of patients (*n* = 155) and samples (*n* = 891).

The PK model chosen for the evaluation was meticulously developed based on a large number of patients and a dense sampling scheme [16]. In addition, the model development dataset originated from the same study center as the evaluation dataset. PK model evaluation revealed only a small bias (−0.84 mg/L) to underpredict observed meropenem concentrations. This small bias was accepted based on its minor size compared to the measured concentrations in our study (mean meropenem concentration 13.0 mg/L) and its tendency to underpredict observed concentrations: the small observed bias leads to more conservative risk predictions and thus adds a further safety margin to the risk assessment [31]. The observed precision (50% prediction error interval ranging from −5.0 mg/L (−59%) to 1.2 mg/L (32%)) was judged to be acceptable for a critically ill patient population based on retrospective data from clinical routines. Compared to a prospective dataset collected in a controlled clinical trial, retrospective data collected during clinical routine is associated with a higher degree of uncertainty [32], which can inflate the observed imprecision in the model evaluation. The comparison of our results to previously published evaluations of meropenem models in critically ill patients further confirms the suitability of the investigated PK model: D’Haese et al. evaluated eight meropenem population PK models in critically ill patients receiving meropenem as continuous infusion [31]. They found a substantial bias (−8.76 to 7.06 mg/L, mean meropenem concentration in the evaluation dataset: 16.3 mg/L) for all of them with the lowest bias being 2.07 mg/L for the model published by Mattioli et al. [33] However, this model did not take into account renal function as a covariate, even though kidney function is considered to be key for the elimination of meropenem and impaired kidney function is regularly present in critically ill patients [34,35]. Furthermore, the imprecision observed by D’Haese using a root mean squared prediction error was higher for all eight models than the observed imprecision in our study (9.9–42.1 mg/L vs. 6.2 mg/L). Therefore, the selected PK model adequately represented the observed clinical routine data and its predictions were used as a benchmark for the evaluation of the MeroRisk-Calculator.

While in general both, the PK model and the MeroRisk-Calculator predicted very similar meropenem concentrations, there was a distinct difference for a subset: for patients with CLCRCG below 50 mL/min, the MeroRisk-Calculator predicted higher concentrations than the PK model. The reason for the discrepancies in predictions for impaired kidney function can be explained by the different mathematical approaches. The MeroRisk-Calculator is based on a linear regression model on a double natural logarithmic scale describing the relation of meropenem concentrations 8 h after standard dose and CLCRCG. Therefore, the predicted minimum concentration of a patient with a CLCRCG approaching zero would be wrongly estimated to be infinite. The linear model of the MeroRisk-Calculator does not separate between renal and non-renal elimination, the latter accounting for up to 27% of the meropenem clearance [36]. When CLCRCG is reduced, the MeroRisk-Calculator predictions behave as if the non-renal part would decrease at the same rate and time. In contrast, for the PK model, a CLCRCG of zero leaves a meropenem clearance of about 20% of the median clearance, which corresponds well to the observed extent of non-renal clearance.

For MIC values below 8 mg/L, differences in risk predictions were small for all patients, since both the MeroRisk-Calculator and the PK model predicted very low risks for patients with CLCRCG below 50 mL/min. Yet, for MIC values greater or equal to 8 mg/L, patients with a CLCRCG below 50 mL/min had a substantial bias, i.e., the MeroRisk-Calculator underestimated the risk compared to the PK model. Even though MICs of 8 mg/L or higher are uncommon and in most cases would lead to a change of antibiotic, the MeroRisk-Calculator should not be used for MIC values greater or equal to 8 mg/L in patients with a CLCRCG of less than 50 mL/min. We have extended the disclaimer of the tool—which already excluded patients with creatinine clearances outside the range of 25–255 mL/min prior to the evaluation—to integrate the knowledge gained from this evaluation. For patients with a CLCRCG above 50 mL/min, the very good agreement shown by the high value of the CCC for the risk of target non-attainment predictions between the PK model and the MeroRisk-Calculator, indicates an interchangeability between both risk predictions. Therefore, the MeroRisk-Calculator can be used to assess the risk of target non-attainment prior to therapy starting with the same confidence as the successfully evaluated PK model. In contrast to the PK model, the MeroRisk-Calculator is usable for the typical healthcare professional, and requires only low computing power and no internet connection.

The newly added possibility to select a pathogen if the MIC value is unknown considerably extends the applicability of the tool, especially since not all hospitals determine MIC values on a regular basis. Due to the changing MIC distributions, an up-to-date version of the MeroRisk-Calculator integrating the latest MIC distributions reported by EUCAST can be found online (https://www.bcp.fu-berlin.de/en/pharmazie/faecher/klinische_pharmazie/arbeitsgruppe_kloft/forschung/MRc, (accessed on 16 April 2021)). The risk assessment for the 155 patients in our dataset and the 74 pathogens currently in the EUCAST database revealed elevated median risks (>10%) for more than 1 out of 4 pathogens (27%). Those pathogens with elevated risks of target non-attainment and an increasing risk for patients with higher CLCRCG highlights the need for an individual risk assessment prior to therapy start. Based on the median risk of target non-attainment in the investigated population, pathogens of the genus Acinetobacter, Pseudomonas and Staphylococcus were especially identified as high-risk pathogens (range of median risk of target non-attainment for these pathogens: 15.0–34.1%, 27.7–48.5%, and 2.1–56.1%, respectively) stressing once more the need for alternative antibiotic drugs or intensified dosing for infections caused by those pathogens.

The presented work has several limitations. First, the evaluation was carried out at the same study center as the data collection for the MeroRisk-Calculator development. This could potentially have had an impact on patient recruitment and the diversity of the patients observed. At the same time, different bioanalytical quantification methods were used in the two studies, which make the transferability of our results to other centers more likely. Furthermore, a wide range of patient characteristics was studied. Nevertheless, to ensure transferability, the presented results should be verified in a next step by clinical data from (a) different study center(s). Second, retrospective data from clinical routine was used for the presented evaluation. While retrospective data from the clinical routine allows for a cost-effective and practical evaluation of a developed tool in a first step, a prospective clinical study as the “gold standard” of evaluation should follow next. How the use of the MeroRisk-Calculator affects the treatment outcome was beyond the scope of this work; future prospective studies could provide important insights in this regard.

## 4. Materials and Methods

### 4.1. Evaluation Strategy for the MeroRisk-Calculator

A retrospective clinical dataset was collected to evaluate the MeroRisk-Calculator in the critically ill target population. This dataset consisted of concentration measurements at variable time points and after different dosing regimens. The MeroRisk-Calculator uses the provided CLCRCG to predict the meropenem concentration 8 h after standard dosing (1 g, 0.5 h infusion, q8h), i.e., at one specific time point. A large proportion of the concentration measurements of the retrospective dataset, however, were taken at different time points (not exactly 8 h after dose) and therefore a direct evaluation, i.e., a comparison of measured concentrations with predicted concentrations by the MeroRisk-Calculator, without censoring most of the data, was not feasible. To utilize the entire available clinical dataset, including all concentrations measured at variable time points, a compartmental PK model was employed. In contrast to the linear regression used in the MeroRisk-Calculator, a compartmental PK model is able to predict a full drug concentration-time profile, i.e., comparison of observed concentrations taken at any time point with the model-predicted concentration is feasible.

**Figure 5:**
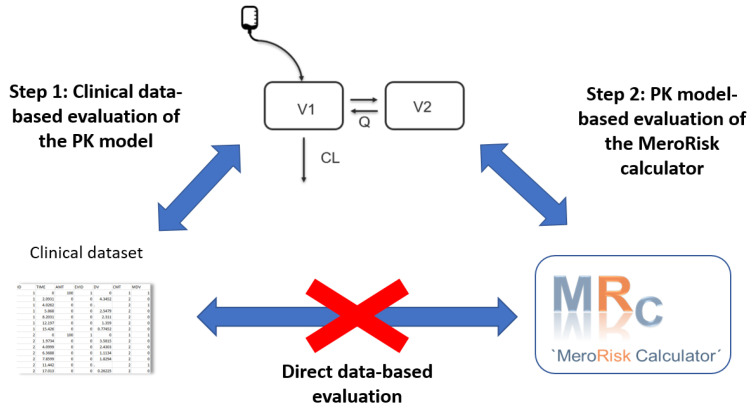
Stepwise evaluation strategy of the MeroRisk-Calculator using a clinical routine dataset. A direct, data-based evaluation of the MeroRisk-Calculator was not feasible due to the time variable sampling time points under routine conditions. A population pharmacokinetic (PK) model was evaluated for its potential to predict the concentrations observed at variable time points (Step 1) and the risk predictions by the PK model were used as a benchmark for the risk predictions of the MeroRisk-Calculator (Step 2).

Overall, a two-step evaluation strategy was employed (Figure 5):

- Step 1: Evaluation of the potential of the selected meropenem population pharmacokinetic (PK) model to predict the clinical routine dataset.
- Step 2: Evaluation of the MeroRisk-Calculator based on PK model predictions of meropenem concentrations 8 h after dosing.

### 4.2. Clinical Data and Patients

Clinical data of patients from a retrospective study at two anaesthesiological intensive care units (ICU) of the University Hospital, LMU Munich, Germany (=evaluation dataset) was collected for the evaluation of the MeroRisk-Calculator. The study protocol (ClinicalTrials.gov identifier: NCT03985605, accessed on: 16 April 2021) was approved by the Institutional Review Board of the Medical Faculty of the LMU Munich (registration number 18–578). All patients received meropenem treatment according to the assessment of the responsible physician. Blood samples were collected daily and meropenem was quantified according to a validated LC-MS/MS method [37]. Demographic patient data (sex, age, weight) and laboratory data (serum albumin concentration, serum creatinine concentration) were collected retrospectively from the hospital information system. Patients with characteristics within the 90% range of the characteristics of the original dataset used for the PK model development (see Section 4.3) were selected for the evaluation. Patients undergoing renal replacement therapy and patients with creatinine clearances outside the range of applicability of the MeroRisk-Calculator (25–255 mL/min) were excluded.

### 4.3. Evaluation Step 1: Evaluation of the Potential of the Selected Meropenem Population Pharmacokinetic (PK) Model to Predict the Clinical Routine Dataset

The model selected to be evaluated was a two-compartment meropenem PK model. It included 3 covariates: CLCRCG as covariate on meropenem clearance, total body weight on the central volume of distribution and serum albumin concentration on the peripheral volume of distribution, implemented as piecewise linear, power and linear relationship, respectively [16].

For the model evaluation of the PK model, 2000 simulations (including interindividual and residual variability) were performed based on the dosing and patient characteristics observed in the evaluation dataset (mrgsolve package (v.0.10.4) in R/Rstudio (v. 3.5.0/v. 1.1.447)). Prediction errors as the difference between observed and predicted meropenem concentrations were calculated and used to assess bias and the precision of the PK model.

### 4.4. Evaluation Step 2: Evaluation of the MeroRisk-Calculator Based on PK Model Predictions of Meropenem Concentrations 8 h after Dosing

For the evaluation of the MeroRisk-Calculator, meropenem concentrations and risks of target non-attainment predicted by the MeroRisk-Calculator (method 1) were compared to those predictions by the evaluated PK model of Step 1 (method 2).

For both methods, total meropenem concentrations at 8 h after standard dose were simulated for the 155 patients included in the evaluation dataset and median predictions were calculated for each patient (PK model: stochastic simulations (*n* = 2000), MeroRisk-Calculator: classic theory of linear models [11]) and compared visually between the methods.

For the risk of target non-attainment analysis, a target of 100% T > MIC, MIC values ranging from 0.125–16 mg/L and the characteristics of the 155 patients of the evaluation dataset were chosen. The risk predictions of the PK model were calculated based on the predicted meropenem concentrations for 2000 virtual patients 8 h after the dose (C8h). For the MeroRisk-Calculator, the risk predictions were derived using the classic theory of linear models and standardized residuals [11]. Lin’s concordance correlation coefficient (CCC)—a common way to calculate the agreement of a new test or measurement (here: MeroRisk-Calculator) to an established test or measurement (here: successfully evaluated PK model)—was chosen to examine the conformity of both predictions [38]. Based on strength-of-agreement criteria for CCC defined by McBride (poor < 0.90, moderate 0.9–0.95, substantial 0.95–0.99, almost perfect > 0.99 [25]) the evaluation of the MeroRisk-Calculator was considered successful, if the lower one-sided 95% confidence limit of the calculated CCC value including all investigated MIC values was larger than 0.95.

### 4.5. Integration of Risk Assessment Based on Pathogen-Specific MIC Distribution

In addition to the risk assessment of target non-attainment for an individual patient (characterized by the CLCRCG) and MIC, a risk assessment based on general pathogen sensitivity data (EUCAST MIC distribution) was implemented into the MeroRisk-Calculator using Excel 2016 software with Visual Basic for Applications (Microsoft Corporation, Redmond, WA, USA). The extended risk calculation was based on cumulative fraction of response analysis (CFR) for the 74 pathogens included in the current report of MIC value distributions by EUCAST [26]. To assess the adequacy of standard dosing for the 155 critically ill patients in the evaluation dataset and all 74 currently in the EUCAST database available pathogens, the risk of target non-attainment was calculated using the new feature of the MeroRisk-Calculator.

## 5. Conclusions

We successfully evaluated the MeroRisk-Calculator using a two-step data- and model-based approach. In comparison to the successfully evaluated compartmental PK model, the MeroRisk-Calculator allows an equally good and reliable but more user-friendly risk assessment for patients with standard meropenem doses and maintained renal function. For patients with CLCRCG < 50 mL/min the tool should not be used. The extension of the MeroRisk-Calculator to include risk assessment based on general pathogen sensitivity data allows a wider range of application and the successful evaluation of the tool proves an appropriate risk assessment in clinical routines. The two-step approach chosen for the evaluation allowed the inclusion of a large number of patients and samples from the clinical routine, thereby increasing robustness of the results without the need for a prospective study.

## Figures and Tables

**Table 1:** Patient characteristics of evaluation dataset.

Patient Characteristic	
Categorical	n (%)
No. of patients No. of male patients No. of meropenem samples	155 101 (65.2) 891
No. of meropenem samples collected during extracorporeal membrane oxygenation	64 (7.18)
Continuous (unit)	Median (5th–95th percentile)
Meropenem concentration (mg/L)	9.05 (1.09–36.5)
Age (years)	57.0 (33.7–79.0)
Weight (kg)	73.0 (50.0–97.3)
Creatinine clearance ^#^ (mL/min)	86.4 (35.4–161)
Serum albumin concentration (g/dL)	2.5 (2.3–3.2)

^#^ Calculated using the Cockcroft–Gault Formula [23]. Meropenem concentration, creatinine clearance and serum albumin concentration determined on sample level, all other continuous characteristics on patient level.

**Table 2:** Lin’s concordance correlation coefficient for risk predictions by pharmacokinetic model and MeroRisk-Calculator.

MIC (mg/L)	Lin’s Concordance Correlation Coefficient (95% CI)	
	All Patients	CLCRCG > 50 mL/min
0.125	0.791 (0.746–0.830) *	0.999 (0.998–0.999) ****
0.25	0.845 (0.811–0.872) *	0.997 (0.996–0.998) ****
0.5	0.894 (0.869–0.914) *	0.992 (0.991–0.994) ****
1	0.921 (0.899–0.938) *	0.930 (0.910–0.946) **
2	0.957 (0.942–0.967) **	0.919 (0.893–0.938) *
4	0.979 (0.972–0.984) ***	0.954 (0.938–0.967) **
8	0.857 (0.834–0.877) *	0.978 (0.970–0.984) ***
16	0.087 (0.077–0.097) *	0.945 (0.925–0.960) **
0.125–16	0.983 (0.981–0.984) ***	0.990 (0.988–0.991) ***

CLCRCG: Creatinine clearance estimated using Cockcroft–Gault Equation [23], Strength of agreement criteria defined by McBride [25]: poor: *, moderate **, substantial ***, almost perfect ****.

## Data Availability

The data presented in this study are available on reasonable request from the corresponding author.

## Supplementary Materials

The following are available online at https://www.mdpi.com/article/10.3390/antibiotics10040468/s1, Figure A1: Model evaluation; Supplementary Materials B: MeroRisk-Calculator; Figures C1–C5: Risk of target non-attainment for all EUCAST-listed pathogens.
