# Supplementary material for: Evaluation of the MeroRisk Calculator, A User-Friendly Tool to Predict the Risk of Meropenem Target Non-Attainment in Critically Ill Patients
Source: Antibiotics (Basel). 2021 Apr 20;10(4):468. doi: 10.3390/antibiotics10040468 (PMC8074046; doi:10.3390/antibiotics10040468)
Supplement: Supplementary file 1 [file antibiotics-10-00468-s001.zip › Appendix_A.pdf]

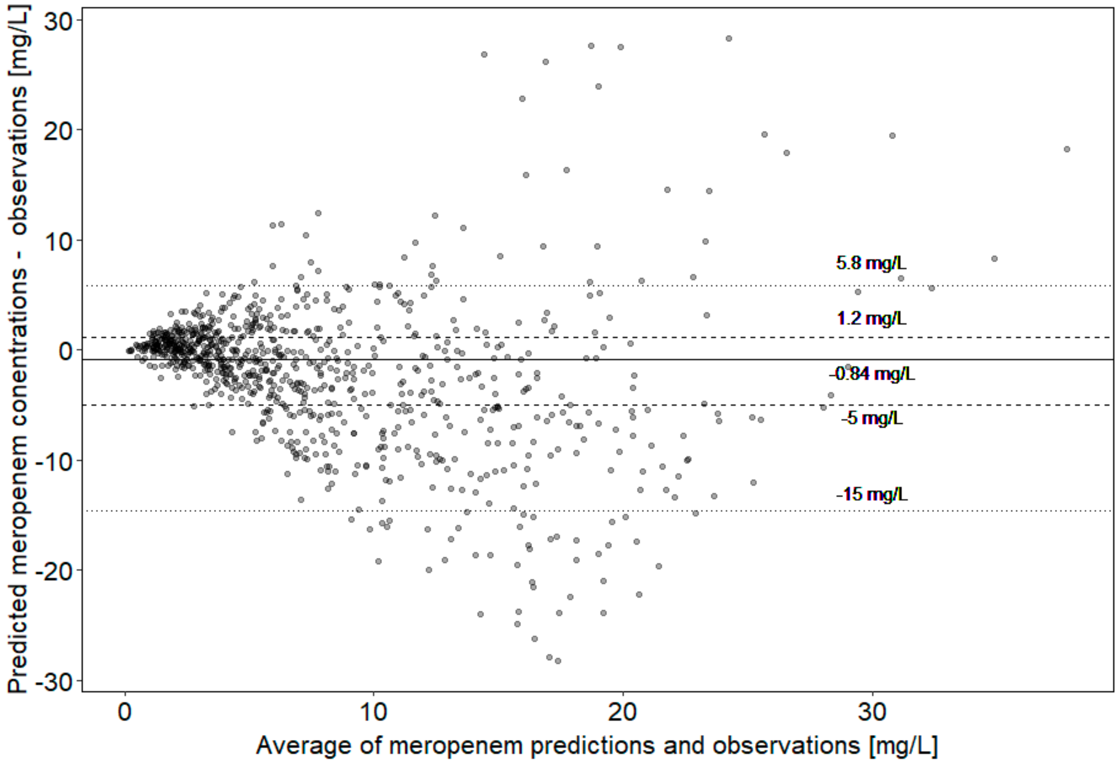

Figure A1: Bland-Altman plot for observed and median predicted meropenem concentrations. Median predictions are based on 1000 stochastic simulations of the investigated pharmacokinetic model. *Solid Line:* median of deviations between observed and median predicted concentrations. *Dashed Lines:* 50% prediction error interval, *Dotted Line:* 90%prediction error interval.
