# Supplementary material for: Evaluation of the MeroRisk Calculator, A User-Friendly Tool to Predict the Risk of Meropenem Target Non-Attainment in Critically Ill Patients
Source: Antibiotics (Basel). 2021 Apr 20;10(4):468. doi: 10.3390/antibiotics10040468 (PMC8074046; doi:10.3390/antibiotics10040468)
Supplement: Supplementary file 1 [file antibiotics-10-00468-s001.zip › Appendix_C.pdf]

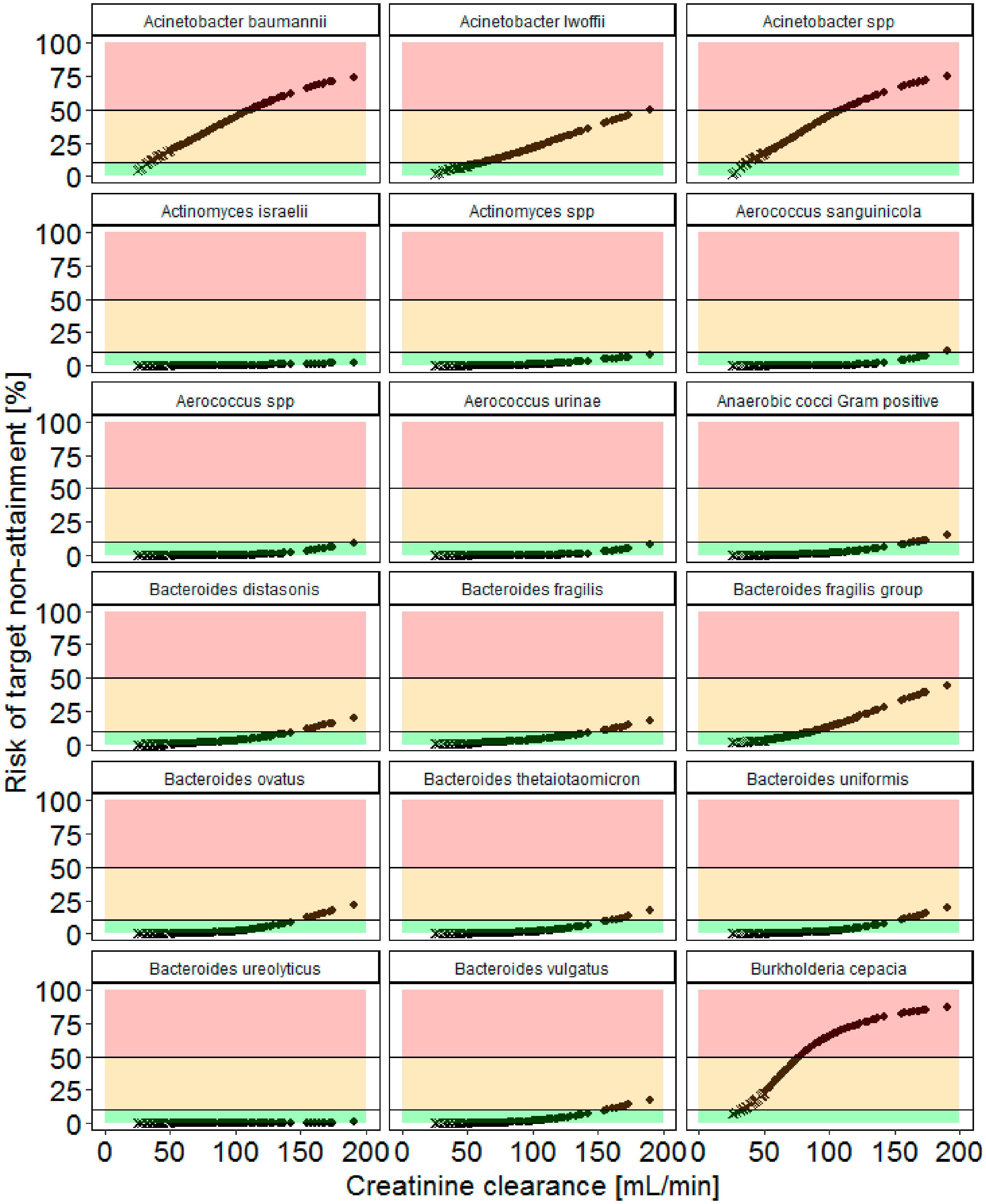

Figure C1: MeroRisk calculator predicted risk of target non-attainment for critically ill patents (n = 155) and alphabetically ordered pathogens from *Acinetobacter baumannii* to *Burkholderia cepacia*. The risk of target non-attainment (drug concentration being above the minimum inhibitory concentration (MIC) for 100% of the time) was assessed using EUCAST MIC distributions of the investigated pathogens and cumulative fraction of response analysis. *Points:* risk predictions for patients with creatinine clearance calculated using Cockcroft-Gault equation (CLCRCG) $\geq$ 50 mL/min. *X:* risk predications for patients with CLCRCG < 50 mL/min. *Green shade:* Risk predictions $\leq$ 10%, *Orange shade:* Risk predictions > 10% to $\leq$ 50%, *Red shade:* Risk predictions > 50%,

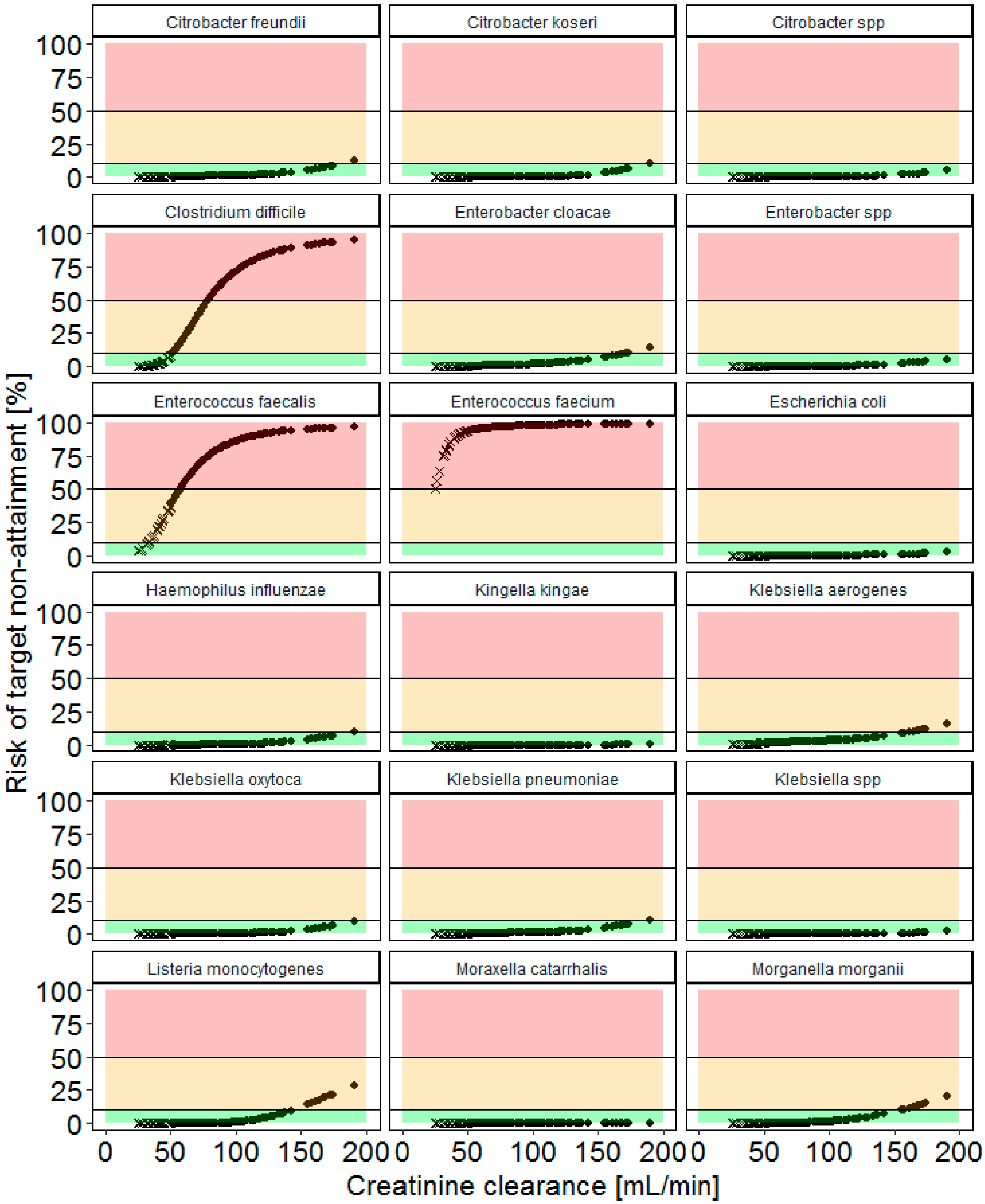

Figure C2: MeroRisk calculator predicted risk of target non-attainment for critically ill patents (n = 155) and alphabetically ordered pathogens from *Citrobacter freundii* to *Morganella morganii*. The risk of target non-attainment (drug concentration being above the minimum inhibitory concentration (MIC) for 100% of the time) was assessed using EUCAST MIC distributions of the investigated pathogens and cumulative fraction of response analysis. *Points:* risk predictions for patients with creatinine clearance calculated using Cockcroft-Gault equation (CLCRCG) > 50 mL/min. *X:* risk predications for patients with CLCRCG < 50 mL/min. *Green shade:* Risk predictions < 10%, *Orange shade:* Risk predictions > 10% to < 50%, *Red shade:* Risk predictions > 50%,

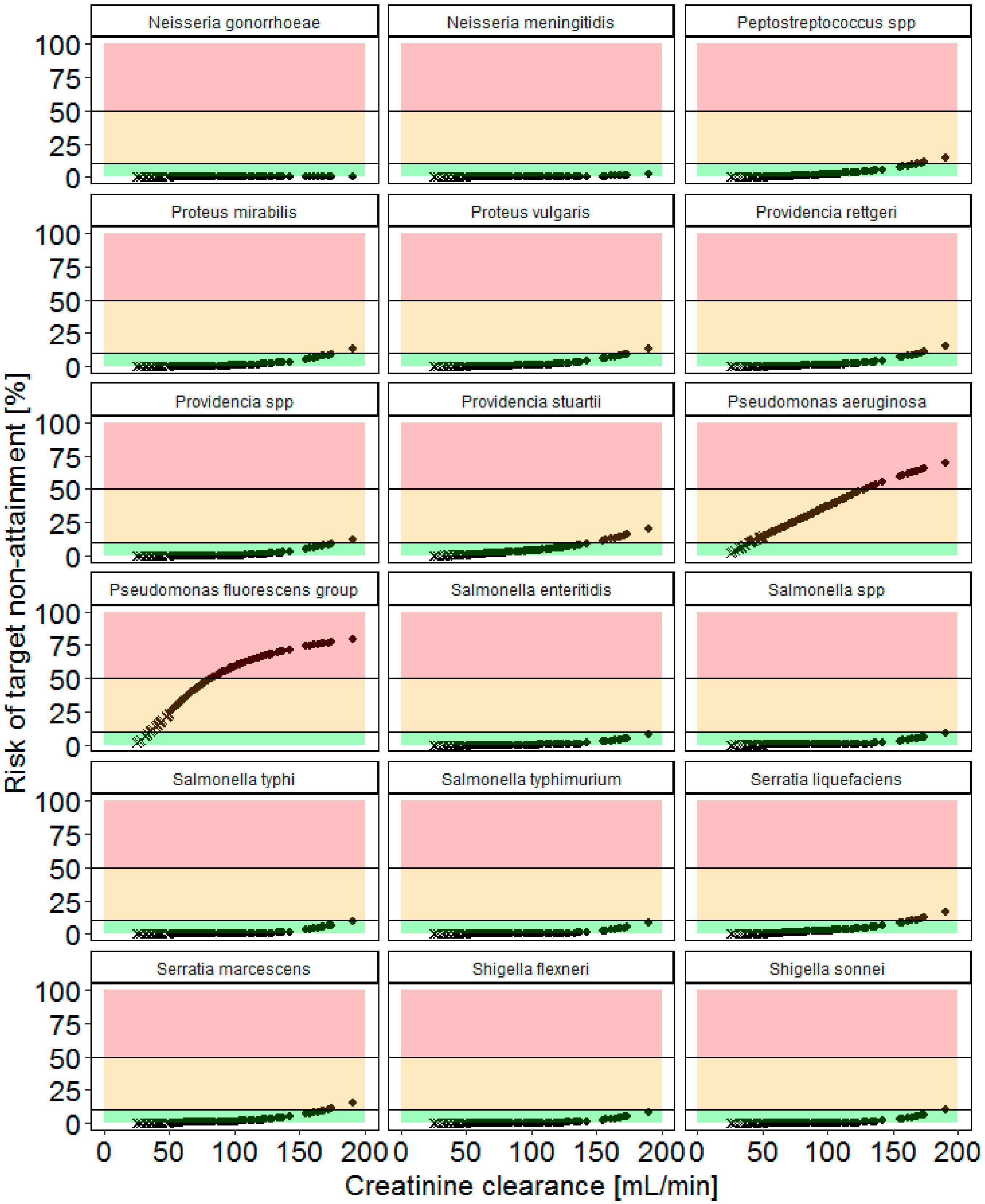

Figure C3: MeroRisk calculator predicted risk of target non-attainment for critically ill patents (n = 155) and alphabetically ordered pathogens from *Neisseria gonorrhoeae* to *Shigella sonnei*.. The risk of target non-attainment (drug concentration being above the minimum inhibitory concentration (MIC) for 100% of the time) was assessed using EUCAST MIC distributions of the investigated pathogens and cumulative fraction of response analysis. *Points:* risk predictions for patients with creatinine clearance calculated using Cockcroft-Gault equation (CLCRCG) ≥ 50 mL/min. *X:* risk predications for patients with CLCRCG < 50 mL/min. *Green shade:* Risk predictions ≤ 10%, *Orange shade:* Risk predictions > 10% to ≤ 50%, *Red shade:* Risk predictions > 50%,

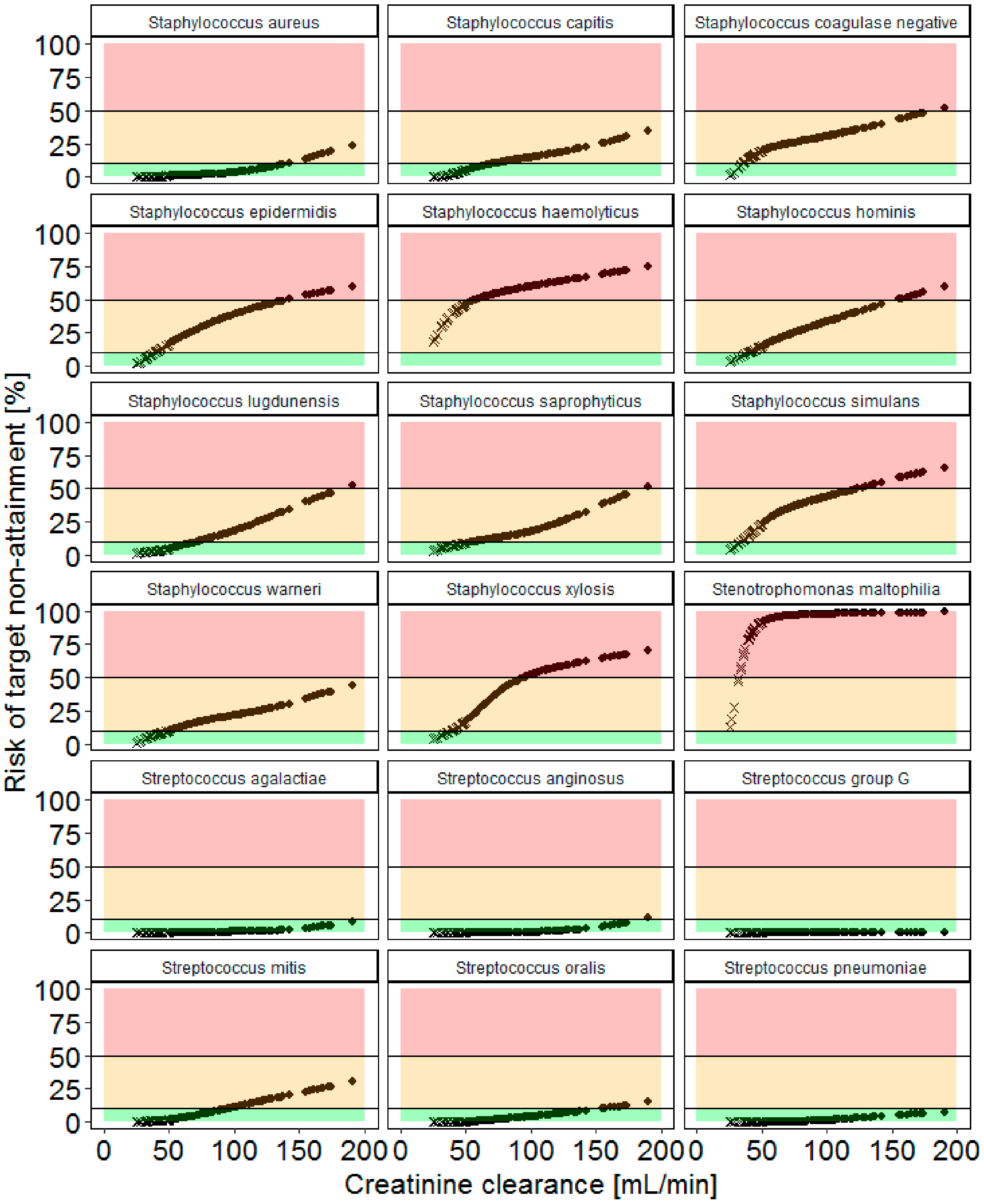

Figure C4: MeroRisk calculator predicted risk of target non-attainment for critically ill patents (n = 155) and alphabetically ordered pathogens from *Staphylococcus aureus* to *Streptococcus pneumoniae*. The risk of target non-attainment (drug concentration being above the minimum inhibitory concentration (MIC) for 100% of the time) was assessed using EUCAST MIC distributions of the investigated pathogens and cumulative fraction of response analysis. *Points:* risk predictions for patients with creatinine clearance calculated using Cockcroft-Gault equation (CLCRCG) ≥ 50 mL/min. *X:* risk predications for patients with CLCRCG < 50 mL/min. *Green shade:* Risk predictions ≤ 10%, *Orange shade:* Risk predictions > 10% to ≤ 50%, *Red shade:* Risk predictions > 50%,

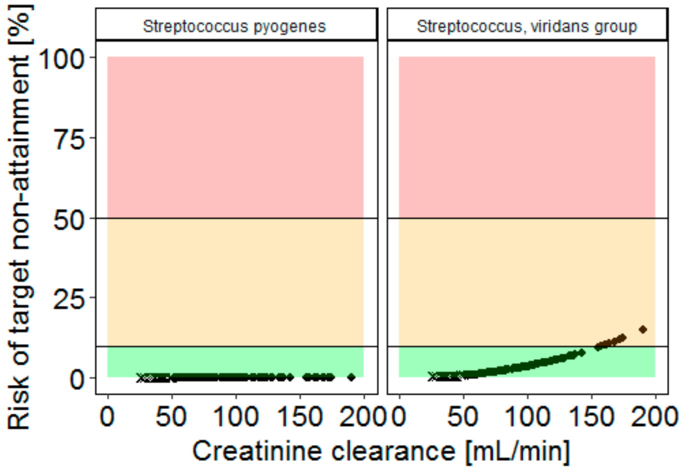

Figure C5: MeroRisk calculator predicted risk of target non-attainment for critically ill patents (n = 155) and *Streptococcus pyogenes* or *Streptococcus viridans group*. The risk of target non-attainment (drug concentration being above the minimum inhibitory concentration (MIC) for 100% of the time) was assessed using EUCAST MIC distributions of the investigated pathogens and cumulative fraction of response analysis. *Points:* risk predictions for patients with creatinine clearance calculated using Cockcroft-Gault equation (CLCRCG) $\geq$50 mL/min. *X:* risk predications for patients with CLCRCG < 50 mL/min. *Green shade:* Risk predictions $\leq$ 10%, *Orange shade:* Risk predictions > 10% to $\leq$ 50%, *Red shade:* Risk predictions > 50%,
